# Low SARS-CoV-2 Seroprevalence and No Active Infections among Dogs and Cats in Animal Shelters with Laboratory-Confirmed COVID-19 Human Cases among Employees

**DOI:** 10.3390/biology10090898

**Published:** 2021-09-11

**Authors:** Caitlin M. Cossaboom, Alexandra M. Medley, Jessica R. Spengler, Esther A. Kukielka, Grace W. Goryoka, Tiffany Baird, Swity Bhavsar, Stefanie Campbell, Thomas S. Campbell, Daniel Christensen, Jillian A. Condrey, Patrick Dawson, Jeffrey B. Doty, Amanda Feldpausch, Julie Gabel, Dee Jones, Ailam Lim, Christina M. Loiacono, Melinda Jenkins-Moore, Andrea Moore, Clarissa Noureddine, Jorge Ortega, Keith Poulsen, Jane A. Rooney, John Rossow, Karen Sheppard, Emma Sweet, Robyn Stoddard, Rachel M. Tell, Ryan M. Wallace, Carl Williams, Casey Barton Behravesh

**Affiliations:** 1 Centers for Disease Control and Prevention, Atlanta, GA 30333, USA; muv3@cdc.gov (A.M.M.); wsk7@cdc.gov (J.R.S.); pgz9@cdc.gov (E.A.K.); lie0@cdc.gov (G.W.G.); Stefanie_campbell@nps.gov (S.C.); nro8@cdc.gov (J.A.C.); wpb7@cdc.gov (P.D.); uwb7@cdc.gov (J.B.D.); dii7@cdc.gov (A.M.); MWI4@cdc.gov (J.R.); frd8@cdc.gov (R.S.); euk5@cdc.gov (R.M.W.); dlx9@cdc.gov (C.B.B.); 2 Epidemic Intelligence Service, Centers for Disease Control and Prevention, Atlanta, GA 30333, USA; 3 Georgia Department of Public Health, Atlanta, GA 30303, USA; tiffany.baird@dph.ga.gov (T.B.); tcampbel@uga.edu (T.S.C.); amanda.feldpausch@dph.ga.gov (A.F.); julie.gabel@dph.ga.gov (J.G.); 4 Guilford County Animal Services, Greensboro, NC 27409, USA; sbhavsar@guilfordcountync.gov (S.B.); cnoureddine@forensivet.com (C.N.); jortega1@guilfordcountync.gov (J.O.); 5 Wisconsin Veterinary Diagnostic Laboratory, Madison, WI 53706, USA; dan.christensen@wvdl.wisc.edu (D.C.); Ailam.Lim@WVDL.wisc.edu (A.L.); Keith.Poulsen@wvdl.wisc.edu (K.P.); emma.sweet@WVDL.wisc.edu (E.S.); 6 Alabama Department of Public Health, Montgomery, AL 36104, USA; dee.jones@adph.state.al.us; 7 United States Department of Agriculture, National Veterinary Services Laboratory, Ames, IA 50010, USA; christina.m.loiacono@usda.gov (C.M.L.); melinda.jenkins-moore@usda.gov (M.J.-M.); rachel.m.tell@usda.gov (R.M.T.); 8 United States Department of Agriculture, Fort Collins, CO 80526, USA; jane.a.rooney@usda.gov; 9 Huntsville Animal Services, Huntsville, AL 35805, USA; karen.sheppard@huntsvilleal.gov; 10 North Carolina Division of Public Health, Raleigh, NC 27699, USA; carl.williams@dhhs.nc.gov

**Keywords:** SARS-CoV-2, COVID-19, zoonosis, coronavirus, human-to-animal, animal shelter

## Abstract

**Simple Summary:**

We investigated dogs and cats living in four animal shelters in the United States that had been exposed to people with COVID-19 in the shelters. Our objective was to understand if the animals were infected with SARS-CoV-2, the virus that causes COVID-19. We found that out of the 96 dogs and cats that we sampled, none had active SARS-CoV-2 infections and only one dog had detectable antibodies to SARS-CoV-2, meaning that it had been exposed to the virus but was not actively infected. This suggests that the risk of humans spreading SARS-CoV-2 to dogs and cats in animal shelter settings is probably low.

**Abstract:**

Human-to-animal and animal-to-animal transmission of SARS-CoV-2 has been documented; however, investigations into SARS-CoV-2 transmission in congregate animal settings are lacking. We investigated four animal shelters in the United States that had identified animals with exposure to shelter employees with laboratory-confirmed COVID-19. Of the 96 cats and dogs with specimens collected, only one dog had detectable SARS-CoV-2 neutralizing antibodies; no animal specimens had detectable viral RNA. These data indicate a low probability of human-to-animal transmission events in cats and dogs in shelter settings with early implementation of infection prevention interventions.

## 1. Introduction

SARS-CoV-2, the virus that causes coronavirus disease 2019 (COVID-19), has spread globally, primarily by human-to-human transmission. Under experimental conditions, SARS-CoV-2-infected cats, ferrets and golden Syrian hamsters exhibited intraspecies transmission to naïve cohabitants [1,2,3]. A small number of animals, including dogs and cats, have been naturally infected with SARS-CoV-2 [4,5,6]. Most natural infections in animals have occurred in domestic or congregate settings such as zoological parks or mink farms where animals have had direct or indirect exposure to people with COVID-19 [7,8,9,10,11]. Transmission from companion animals to humans has not been documented. It is not known what level of human-to-animal transmission may occur in other settings where human–animal interactions are frequent but may differ in nature from those in domestic household settings. Specifically, investigations to characterize the risk of human-to-animal SARS-CoV-2 transmission in congregate animal settings, such as animal shelters, are lacking.

Beginning in May 2020, the U.S. Centers for Disease Control and Prevention (CDC), in partnership with state and local governments and the U.S. Department of Agriculture (USDA) Animal and Plant Health Inspection Service, Veterinary Services, worked with state and local governments to conduct active surveillance for SARS-CoV-2 infection in animals residing in animal shelters that had identified employees or volunteers with laboratory-confirmed COVID-19 that may have exposed animals within their facilities to SARS-CoV-2. Therefore, we sought to: (1) investigate SARS-CoV-2 infection and seroprevalence among dogs and cats in animal shelters with exposure to shelter staff with laboratory-confirmed COVID-19; (2) characterize the potential risk factors for human-to-animal transmission; and (3) describe COVID-19 infection prevention and control precautions implemented in animal shelter settings.

## 2. Materials and Methods

Four shelters in the United States, two in Georgia (GA-01 and GA-02) and one each in Alabama (AL-01) and North Carolina (NC-01), were enrolled in this study between May–July 2020. During this time, the United States was experiencing a surge in COVID-19 cases nationwide. From 1 May to 31 July 2020, confirmed COVID-19 cases increased from approximately 27.5 to 186.4 thousand in Georgia, 7.4 to 88.3 thousand in Alabama and 11.6 to 125.7 thousand in North Carolina [12]. Animal shelters were enrolled in this study contingent on confirmation that close contact occurred between at least one animal and one person with laboratory-confirmed COVID-19 within the facilities either during the symptomatic period or during the two days prior to a positive SARS-CoV-2 test for an asymptomatic person [13]. Close contact and exposure were defined as being within approximately six feet (two meters) of a person with confirmed COVID-19 for at least fifteen minutes or having direct contact with infectious secretions from a person with confirmed COVID-19 (e.g., being coughed on, consuming food or objects contaminated with a human patient’s mucous or saliva) [13]. Exposure periods were calculated beginning two days prior to positive specimen collection of the human COVID-19 cases and ended the last day the person had animal contact in the facility before self-isolating.

Specimen collection was conducted on one day at each of three shelters (GA-01, GA-02 and AL-01) and over three days at one shelter (NC-01). Timing of specimen collection post-exposure varied and was reliant on the timing of the initial identification of the human cases within the facilities and approval of the field investigation by state officials; field teams began animal investigations immediately following approval. Five diagnostic specimens for laboratory-confirmation of SARS-CoV-2 infection were collected from all animals: nasal, oral and rectal swabs and fecal and blood specimens [14]. Fur swab specimens were also collected to assess environmental contamination on the animals (Appendix A). Swab and fecal specimens were tested by a modified SARS-CoV-2 rRT-PCR based on published CDC 2019-nCoV assay [15] following ribonucleic acid extraction; blood specimens were tested for SARS-CoV-2 serum virus neutralization (Appendix A). Both a facility-level questionnaire and an individual animal questionnaire (Figures S1 and S2) were administered to representatives of each shelter facility. The facility questionnaire contained questions regarding staff and animals in the facility, including animal interactions with staff with confirmed COVID-19 and infection prevention protocols in place prior to and post exposure. The individual animal questionnaire was completed for each exposed animal and contained questions about the animal’s history prior to arrival in the shelter, medical history, daily routine, housing and SARS-CoV-2 exposure history within the shelter.

## 3. Results

In total, 96 animals were enrolled among the four participating shelters, including 54 (56%) cats and 42 (44%) dogs. Specimens were collected from 47 animals in GA-01, 7 in GA-02, 30 in NC-01 and 12 in AL-01 (Table 1). Five diagnostic specimens (nasal, oral and rectal swabs and fecal and blood specimens) were collected from all animals except for a small number that could not be collected due to reasons including demeanor of the animal or unavailability (Table 1). Among the 96 animals across the four shelters, 702 total specimens were collected, including 120 nasal swabs, 92 oropharyngeal swabs, 95 rectal swabs, 65 fecal specimens, 91 blood specimens and 148 fur swabs (Table 1). Average age of the animals by facility was 1.5 years in GA-01, 2.3 years in GA-02, 4.1 years in NC, and 3.8 years in AL; overall average age was 2.6 years and 51% of animals were male. Ninety-two (96%) of the sampled animals were exposed to an employee with confirmed COVID-19 and fit the study definition of exposed; four animals were co-housed with other exposed animals but were taken in after the exposure occurred (Table 2). Most (57%) animals were stray/loose when they were taken into the shelters (Table 2). Duration of exposure of shelter animals to people with confirmed COVID-19 was typically <1 h (Table 2). Type of interaction varied widely; petting/cuddling, cleaning the animal’s housing, going for walks or play yards and feeding were most common (Table 2). Fifteen (15.6%) animals developed non-specific respiratory or gastrointestinal signs between the date of exposure to a person with COVID-19 and specimen collection.

Of the 372 diagnostic specimens (including nasal, oropharyngeal, rectal and fecal specimens) collected from 96 animals across the four participating shelters, none tested positive by SARS-CoV-2 rRT-PCR. Likewise, all fur swabs tested negative by SARS-CoV-2 rRT-PCR. Of the 90 animals with blood specimens collected across the four shelters, one asymptomatic dog in the NC shelter had detectable SARS-CoV-2 neutralizing antibodies (1:32 titer); a repeat blood specimen collected two weeks after initial collection yielded the same titer. The seropositive dog had been living in the shelter for six weeks prior to specimen collection and had similar interactions with the three persons with confirmed COVID-19 as all other exposed animals in the NC shelter. The dog’s SARS-CoV-2 exposure history prior to shelter entry is unknown.

**Figure 1:**
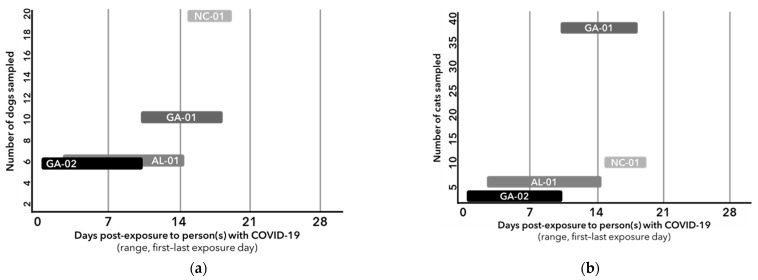
Timelines depicting number of dogs (**a**) and cats (**b**) sampled by days post-exposure to person(s) with confirmed COVID-19 during an investigation of SARS-CoV-2 transmission in congregate animal settings in USA, 2020.

The number of shelter employees with confirmed COVID-19 ranged from two to four at any given location. The average calculated exposure periods for animals across the four shelters was 6.8 days (range: 4–11). Timing of specimen collection relative to exposure period varied by animal within each shelter. In each shelter, the range of days following the exposure period (first to last reported exposure) to person(s) with confirmed COVID-19 during which specimens were collected from exposed animals were 15–19 days in NC-01, 3–14 days in AL-01, 9–17 days in GA-01 and 1–10 days in GA-02. Timelines were constructed to depict the number of dogs and cats sampled by days post-exposure to person(s) with confirmed COVID-19 in each shelter (Figure 1).

Prior to the identification of staff with confirmed COVID-19, all four shelters had developed and implemented plans for continuity of essential functions during the pandemic, including COVID-19 infection prevention and personal protective equipment (PPE) training. Staff were required to wear cloth face coverings or disposable surgical-style masks inside the facility and disposable gloves when handling animals. Staff underwent daily pre-shift health screening; anyone with symptoms consistent with COVID-19 returned home to self-isolate. The shelters had also temporarily restricted access to certain areas of the facility to some staff; adoptions were by appointment only.

## 4. Discussion

All swab and fecal specimens collected from the 96 animals exposed to persons with COVID-19 across the four shelter investigations tested negative by SARS-CoV-2 rRT-PCR. Of the 90 animals with blood specimens collected, one asymptomatic dog in the NC shelter had detectable SARS-CoV-2 neutralizing antibodies (1:32 titer); a repeat blood specimen collected two weeks later yielded the same titer. Shelter-wide re-sampling was deemed unnecessary as there was no evidence of continued SARS-CoV-2 transmission within the facility. For the seropositive dog, the stable convalescent titer, six-week shelter confinement and history of exposure to staff with confirmed COVID-19 suggests that asymptomatic infection and seroconversion probably occurred due to exposure at the shelter. These findings suggest that human-to-animal transmission in animal shelter settings is possible but rare when appropriate infection prevention precautions are instituted.

It is important to note limitations of these investigations. First, timing of specimen collection post-exposure varied by animal and facility and was dependent on the timing of the initial identification and notification of the human cases and approvals of the field investigations. Animals were sampled 9–19 days following the last known exposure to any person with confirmed COVID-19 (Figure 1); viral shedding may have been missed if animals cleared the virus prior to specimen collection. SARS-CoV-2 neutralizing antibody response in animals is not well described; however, in humans it appears variable following clinical recovery [16]. Staff with confirmed SARS-CoV-2 in the four shelters reportedly wore appropriate PPE and had minimal prolonged direct contact with animals. This may not be representative of shelters throughout the U.S.; settings implementing fewer infection prevention interventions, or where human-to-animal contact is more frequent or of longer duration, may pose a higher risk for SARS-CoV-2 transmission to animals. Additionally, 15 of 96 (15.6%) animals developed non-specific respiratory or gastrointestinal signs following exposure, which were probably due to more common etiologies that are frequently reported in shelter settings unrelated to SARS-CoV-2; however, no differential testing was performed, and shelters followed their standard treatment protocols.

In 2020, there were over 2.6 million cats and dogs residing in animal shelters in the U.S. that interacted daily with shelter employees and volunteers [17]. Previous studies that have assessed seroprevalence in companion animals living in households with human COVID-19 cases have identified seropositivity up to 43.8% in cats and 20% in dogs [7,8,9,18,19,20]. In our investigations in four animal shelters in the U.S., infection prevention interventions had been implemented to reduce the risk of human-to-human and human-to-animal SARS-CoV-2 transmission. None of the specimens collected from 96 animals across the four shelters tested positive by SARS-CoV-2 rRT-PCR, and only one dog was seropositive by virus neutralization assay. Taken together, our findings suggest that these mitigation strategies were successful and that there is a low likelihood of human-to-animal transmission in animal shelter settings where human–animal interactions are less frequent and shorter in duration than in domestic household settings. Further investigation is needed to better understand risk factors for human-to-animal and animal-to-animal spread of SARS-CoV-2, particularly in congregate settings such as animal shelters. These investigations are critical to inform guidance for SARS-CoV-2 prevention and control strategies for both people and animals in these and other congregate settings.

## Figures and Tables

**Table 1:** Summary of enrolled animals and specimens collected from each of the four participating animal shelters during an investigation of SARS-CoV-2 transmission in congregate animal settings in USA, 2020.

Shelter ID	Total Animals *n* = 96	Animal Species		Total Specimens *n* = 702	Specimen Type					
		Dog *n* = 42	Cat N = 54		Nasal Swabs * *n* = 120	OP Swabs * n = 92	Rectal Swabs * n = 95	Fecal Specimens * n = 65	Blood Specimens * n = 91	Fur Swabs *n* = 148
GA-01	47	10	37	292	47	45 †	47	30 †	38 †	47
GA-02	7	6	1	41	7	7	6†	0 †	7	7
AL-01	12	6	6	85	12	12	12	7 †	12	12
NC-01	30	20	10	284	54 †,‡	28	30	28	31 §	82 ‡

* Diagnostic specimens for laboratory confirmation of SARS-CoV-2 infection include nasal, oral and rectal swabs and fecal specimens tested by SARS-CoV-2 rRT-PCR and blood/sera specimens tested by SARS-CoV-2 virus neutralization. † Not all specimen types were able to be collected from all animals due to reasons including demeanor of the animal or unavailability (in the case of fecal specimens). ‡ In the NC shelter investigation, nasal and fur swabs were collected in duplicate. § In the one seropositive dog in the NC shelter investigation, a second blood specimen was collected two weeks after the first.

**Table 2:** Characteristics of intake, history of SARS-CoV-2 human-to-animal exposure(s) and animal housing during an investigation of SARS-CoV-2 transmission in congregate animal settings in USA, 2020.

	Overall (*n* = 96)
**Reason for intake of the animal**	
Owner surrender unrelated to COVID-19	21 (21.9%)
Stray/loose animal	55 (57.3%)
Bite hold	3 (3.1%)
Returned by adopter or foster	1 (1.0%)
Transferred from another facility (e.g., animal shelter)	16 (16.7%)
**Source of exposure of the animal to SARS-CoV-2**	
Facility employee (paid or unpaid)	92 (95.8%)
No known exposure *	4 (4.0%)
**Daily duration of the interaction between the animal and the person(s) with confirmed COVID-19**	
<1 h	51 (53.1%)
1–3 h	5 (5.2%)
Unknown	6 (6.3%)
No known exposure *	4 (4.0%)
Data not collected (North Carolina shelter)	34 (35.4%)
**Types of interaction the person(s) with confirmed COVID-19 had with the animal**	
Petting/cuddling	54 (56.3%)
Cleaning the animal’s housing	51 (53.1%)
Going for walks or play yards	49 (51.0%)
Feeding	50 (52.1%)
Play groups with other animals	35 (36.5%)
Veterinary care	7 (7.3%)
Indirect—within 6 feet but no direct contact	6 (6.3%)
Behavioral assessments or training	3 (3.1%)
In staff member’s office full time	2 (2.1%)
Grooming	1 (1.0%)
Picked up	1 (1.0%)
Transport, bringing into intake	1 (1.0%)
**Quarantine setting for the animal(s) exposed to SARS-CoV-2**	
Yes, quarantine area for animals exposed to SARS-CoV-2 or with other medical conditions or exposures	3 (3.1%)
No	93 (96.9%)
**Type of animal interaction with other animals (e.g., dogs participating in play group)**	
Play group	4 (4.2%)
Goes out to the yard	1 (1.0%)
Interacts with other outdoor cats on shelter property	1 (1.0%)
No interaction with other animals	1 (1.0%)
Unknown	89 (92.7%)
**Animal’s current housing**	
Housed individually, with other animals in the same room (e.g., a kennel with multiple, single-animal enclosures in one room)	61 (63.5%)
Co-housed with other animals (e.g., communal cat room, multiple dogs in one run)	30 (31.3%)
Housed individually, no other animals in the room (e.g., in a staff member’s office)	3 (3.1%)
Free-roaming outdoors (community cats)	1 (1.0%)
Other, unspecified	1 (1.0%)

* Refers to animals with no known exposure to people with COVID-19, but the animals were housed with or otherwise exposed to animals with known exposures.

## Data Availability

Data is contained within the article or supplementary material.

## Supplementary Materials

The following are available online at https://www.mdpi.com/article/10.3390/biology10090898/s1: Figure S1: Facility Questionnaire for Animal Shelters with Animals Exposed to Persons with COVID-19, Figure S2: Individual Animal Questionnaire for Shelter Animals Exposed to Persons with COVID-19.

## Appendix A

### Appendix A.1. Animal Specimen Collection

In the North Carolina shelter investigation, fur swabs were collected in duplicate using 2 in × 2 in sterile gauze pads and rubbing across the dorsal and ventral surfaces and around the paw pads of the animal; one swab was stored dry and one in RNAlater (Thermo Fisher Scientific, Waltham, MA, USA). In the remaining three investigations, one fur swab was collected using elongated sterile polyester flock-tipped swabs with 6” polystyrene handles (Puritan Medical Products, Guilford, ME) and stored in 3 mL brain heart infusion (BHI) media. In all investigations, diagnostic specimens (oropharyngeal, nasal and rectal swabs), convenience fecal specimens and up to 3 mL of venous blood were collected. Oropharyngeal, nasal and rectal swabs and fecal specimens were collected and stored in 3 mL BHI media. Blood specimens were centrifuged, and sera were extracted and stored at −80 °C until testing. All animal procedures were approved by the CDC Institutional Animal Care and Use Committee (3104BARMULX).

### Appendix A.2. Ribonucleic Acid Extraction and rRT-PCR

Preliminary ribonucleic acid (RNA) extraction and real-time reverse–transcription polymerase chain reaction (rRT-PCR) testing of animal specimens occurred at the Wisconsin Veterinary Diagnostic Laboratory (Madison, WI, USA). Fur swabs were removed from RNAlater and resuspended in 5 mL of phosphate buffered saline (PBS). Fecal swabs were obtained from fecal specimens and suspended in 1 mL PBS. RNA was extracted from 50 µL of specimen (in BHI or PBS) along with Xeno RNA (10,000 copies, Thermo Fisher Scientific) using the MagMAX-96 Viral RNA Isolation Kit (Thermo Fisher Scientific) on a 96-well KingFisher extraction platform and eluted in a volume of 50 µL according to manufacturer’s instructions. RNA (5 µL) was quantitated using a one-step rRT-PCR targeting the 2019-nCoV nucleocapsid (N) gene (N1 and N2). The rRT-PCR assay was based on the CDC assay (11) with alternative reagents using the 2019-nCoV RUO Kit (Integrated DNA Technologies, Catalog # 10006713), the AgPath-ID One-Step RT-PCR Reagents and the VetMAX Xeno Internal Positive Control (Thermo Fisher Scientific). The rRT-PCR amplification was performed with one cycle at 50 °C for 15 min and 90 °C for 10 min, 40 cycles of 95 °C for 15 s and 55 °C for 1 min on an Applied Biosystems 7500 Fast Real-Time PCR Instrument.

### Appendix A.3. Virus Neutralization Assay

SARS-CoV-2 neutralizing antibody titers were determined at USDA National Veterinary Services Laboratories using a virus neutralization (VN) assay. For VN, 25 µL of two-fold serially diluted sera (from 1:8 to 1:512) were pre-incubated with 25 µL of 100 TCID50/mL of SARS-CoV-2 (2019-nCoV/USA-WA1/2020) in minimal essential medium containing 200 UI/mL penicillin, 200 µg/mL streptomycin, 75 µg/mL gentamicin sulfate and 6 µg/mL Amphotericin B for 60 min at 37 °C with 5% CO2. Each serum specimen was tested in duplicate in 96-well plates. At 1 h post infection, 150 µL of Vero 76 cells was added to the virus–serum mixtures. The neutralization titers were determined at 3 days post infection. The titer of a specimen was recorded as the reciprocal of the highest serum dilution that provided at least 100% neutralization of the reference virus as determined by visualization of cytopathic effect. A neutralizing titer of <32 was considered suspect in the absence of other positive tests (PCR or virus isolation).
