# Supplementary material for: Low SARS-CoV-2 Seroprevalence and No Active Infections among Dogs and Cats in Animal Shelters with Laboratory-Confirmed COVID-19 Human Cases among Employees
Source: Biology (Basel). 2021 Sep 11;10(9):898. doi: 10.3390/biology10090898 (PMC8467101; doi:10.3390/biology10090898)
Supplement: Supplementary file 1 [file biology-10-00898-s001.zip › biology-1352454-supplementary.pdf]

Figure S1: Facility Questionnaire for Animal Shelters with Animals Exposed to Persons with COVID-19

Instructions: A facility-level questionnaire should be completed by an adult (≥ 18 years old) representative from each facility that houses animals in a congregate setting and has identified a COVID-19 positive human case

Facility ID: _________

Date interview completed: / / (MM/DD/YYYY)
Type of interview: ☐ On-site (in person) ☐ Off-site (by phone) ☐ Other (specify): ______________________________
Interviewer name and organization: ______________________________
Respondent's name: ______________________________
Respondent's position at the facility: ______________________________
Respondent's phone number: ______________________________

Instructions: All questions and answers in this survey will remain confidential. I will begin by asking you some questions related to the facility. Please answer to the best of your ability.

1. Facility name: ______________________________
2. Type of facility: ☐ County, city, or state-funded animal services facility ☐ Privately funded animal shelter, rescue, or sanctuary ☐ Animal rehabilitation facility ☐ Animal breeding facility ☐ Animal distribution facility ☐ Pet retail store ☐ Zoological park ☐ Other (please describe) ______________________________
3. Total number of employees (paid or non-paid) at facility: ___________
4. Total number of volunteers at facility: ___________
5. Total number of employees (paid or non-paid) that have contact with animals in the facility on a given day: ______
6. Total number of volunteers that have contact with animals in the facility on a given day: __________
7. How many animals of each species are currently being housed within the facility?
   a. Dogs: ______
   b. Cats: ______
   c. Ferrets: ______
   d. Rabbits: ______
   e. Small Mammals: ______
   f. Livestock (horses, cattle, pigs, small ruminants): ______
   g. Other (please describe): ______________________________
8. What is the maximum animal holding capacity within the facility?
   a. Dogs: ______
   b. Cats: ______
   c. Ferrets: ______
   d. Rabbits: ______
   e. Small Mammals: ______
   f. Livestock (horses, cattle, pigs, small ruminants): ______
   g. Other (please describe): ______
9. Is there a veterinarian on staff at the facility? ☐ Yes, full-time ☐ Yes, part-time ☐ No ☐ Other (please describe) ______________________________
10. Is there a veterinary clinic at the facility? ☐ Yes, full clinic that will see both shelter and client-owned animals ☐ Yes, only for shelter animals ☐ No ☐ Other (please describe) ______________________________
11. Does the facility routinely run diagnostic testing for respiratory and gastrointestinal pathogens?
    ☐ Yes ☐ No ☐ Don't know ☐ Not applicable
    If yes, describe: ______________________________
12. Has the facility had cases of undiagnosed respiratory or gastrointestinal diseases within since the last known date that animals may have been exposed to a COVID-19 human case in the facility?
    ☐ Yes ☐ No ☐ Don't know ☐ Not applicable
    If yes, describe: ______________________________

Instructions: Now I will ask you some COVID-19 related questions about how your facility has addressed and managed COVID-19. As a reminder, all questions and answers in this survey will remain confidential. Please answer to the best of your ability.

"Public reporting burden of this collection of information is estimated to average 10 minutes per response, including the time for reviewing instructions, searching existing data sources, gathering and maintaining the data needed, and completing and reviewing the collection of information. An agency may not conduct or sponsor, and a person is not required to respond to a collection of information unless it displays a currently valid OMB control number. Send comments regarding this burden estimate or any other aspect of this collection of information including suggestions for reducing this burden to CDC/ATSDR Reports Clearance Officer; 1600 Clifton Road NE, MS D-74 Atlanta, Georgia 30333; ATTN: PRA (0920-1011)."

13. Of the persons with COVID-19 that potentially exposed animals in your facility, were they:
    a. Employees (paid or non-paid): ______
    b. Volunteers: ______
    c. Other visitors to the facility: ______
    d. How many of these people had direct animal contact? __________
    e. When was the first date of likely animal exposure to COVID-19 within the facility? ___________
    f. When was the last date of likely animal exposure to COVID-19 within the facility? ____________
14. Of the persons suspected with COVID-19 (symptoms consistent with COIVD-19 with or without pending testing) that potentially exposed animals in your facility, were they:
    g. Employees: ______
    h. Volunteers: ______
    i. Other visitors to the facility: ______
    j. How many total people who potentially exposed animals in the facility have pending tests? ________
    k. When was the first date of likely animal exposure? _________
    l. When was the last date of likely animal exposure within the facility? ____________

Instructions. The following questions relate to the COVID-19 workplace health and safety plan for the facility, please answer to the best of your ability,. reflecting the policies in place during the time period in which exposures occurred

15. Plans have been developed to continue essential functions with a reduced workforce: ☐ Yes ☐ No ☐ Don't know
16. Pre-shift health screening (ex. temperature or symptom check) has been considered or implemented:
    ☐ Yes, considered: ☐ Yes, considered ☐ Yes, implemented ☐ No ☐ Don't know
17. Visitor health screening has been considered or implemented:
    ☐ Yes, considered ☐ Yes, implemented ☐ No ☐ Don't know
18. Policy in place for managing workers with potential exposure to COVID-19: ☐ Yes ☐ No ☐ Don't know
    If yes, describe: ____
19. Policy in place for managing workers with COVID-19: ☐ Yes ☐ No ☐ Don't know
    If yes, what are plans for workers who:
    a. Are ill prior to coming to work? ____________________________________
    b. Are ill at pre-shift screening? ____________________________________
    c. Become ill while at work? ____________________________________
    d. Test positive, regardless of symptoms? ____________________________________
20. Leave policies are flexible, non-punitive, and encourage ill employees to stay home: ☐ Yes ☐ No
    ☐ Other, please describe: ____________________
21. Policy in place for managing return-to-work for workers with COVID-19: ☐ Yes, symptom based ☐ Yes, time based
    ☐ Yes, test based ☐ No ☐ Don't know
    If yes, describe: ____
22. Standard operating procedures for cleaning, disinfection, and sanitation in the facility have been reviewed and modified as necessary for COVID-19: ☐ Yes ☐ No ☐ Don't know ☐ Not applicable
    If yes, describe: ____
    If yes, has this been distributed to employees and/or volunteers for awareness? ☐ Yes, employees ☐ Yes, volunteers
    ☐ No ☐ Don't know ☐ Not applicable
23. Restriction on public access to the facility (e.g., appointment only): ☐ Yes ☐ No ☐ Don't know ☐ Not applicable
    If yes, describe: ____________________________________
24. Modified adoption procedures: ☐ Yes ☐ No ☐ Don't know ☐ Not applicable
    If yes, describe: ____________________________________
25. Modified intake procedures for surrendered animals: ☐ Yes ☐ No ☐ Don't know ☐ Not applicable
    If yes, describe: ____________________________________
26. When a person surrender an animal to the facility, do staff ask if the animal has been exposed to the virus that causes COVID-19? ☐ Yes ☐ No ☐ Don't know ☐ Not applicable
27. If an animal is reported to have had an exposure to the virus that causes COVID-19, either prior to intake or while within the facility, are there modified procedures in place for intake and housing of this animal? ☐ Yes ☐ No ☐ Don't know ☐ Not applicable

"Public reporting burden of this collection of information is estimated to average 10 minutes per response, including the time for reviewing instructions, searching existing data sources, gathering and maintaining the data needed, and completing and reviewing the collection of information. An agency may not conduct or sponsor, and a person is not required to respond to a collection of information unless it displays a currently valid OMB control number. Send comments regarding this burden estimate or any other aspect of this collection of information including suggestions for reducing this burden to CDC/ATSDR Reports Clearance Officer; 1600 Clifton Road NE, MS D-74 Atlanta, Georgia 30333; ATTN: PRA (0920-1011)."

If yes, describe: ____

28. Is there an area designated for animals exposed to SARS-CoV-2 (e.g., isolation or quarantine room)?
☐ Yes, this area only houses animals exposed to SARS-CoV-2
☐ Yes, this is a general quarantine area that houses animals exposed to SARS-CoV-2 as well as animals with other medical conditions or exposures
☐ No ☐ Don't know ☐ Not applicable *If yes, describe*: ____
29. Are there areas within the facility where animals are able to co-mingle with each other (e.g. a waste elimination area, play area)? ☐ Yes, outdoor waste elimination area ☐ Yes, outdoor play area ☐ Yes, indoor area ☐ No
If yes, describe: ____
30. How often are animal housing (cages, kennels, runs) disinfected per day? ☐ Less than once per day ☐ Once a day ☐ Twice a day ☐ Three or more times a day
    a. *If yes, what disinfectant is used (include concentration, if available)?* _________________
31. Are employees (paid or non-paid) required to wear any of the following while they are in the facility?
    b. Cloth face coverings or disposable surgical-style masks: ☐ Yes ☐ No ☐ Don't know
    If yes, when is this required: ____
    c. Respirator (ex. N95): ☐ Yes ☐ No ☐ Don't know
    If yes, when is this required: ____
    d. Eye protection: ☐ Yes ☐ No ☐ Don't know
    If yes, when is this required: ____
    e. Disposable gloves: ☐ Yes ☐ No ☐ Don't know
    If yes, when is this required: ____
    f. Gown or coveralls: ☐ Yes ☐ No ☐ Don't know
    If yes, when is this required: ____
    g. Rubber boots or shoe covers: ☐ Yes ☐ No ☐ Don't know
    If yes, when is this required: ____
    h. Other: ☐ Yes ☐ No ☐ Don't know
    If yes, describe: ____
32. If required, are cloth face coverings and PPE being provided to employees? ☐ Yes ☐ No ☐ Not applicable
33. Are volunteers required to wear any of the following while they are in the facility?
    i. Cloth face coverings or disposable surgical-style masks: ☐ Yes ☐ No ☐ Don't know
    If yes, when is this required: ____
    j. Respirator (ex. N95): ☐ Yes ☐ No ☐ Don't know
    If yes, when is this required: ____
    k. Eye protection: ☐ Yes ☐ No ☐ Don't know
    If yes, when is this required: ____
    l. Disposable gloves: ☐ Yes ☐ No ☐ Don't know
    If yes, when is this required: ____
    m. Gown or coveralls: ☐ Yes ☐ No ☐ Don't know
    If yes, when is this required: ____
    n. Rubber boots or shoe covers: ☐ Yes ☐ No ☐ Don't know
    If yes, when is this required: ____
    o. Other: ☐ Yes ☐ No ☐ Don't know *If yes, describe:* ____
34. If required, are cloth face coverings and PPE being provided to volunteers? ☐ Yes ☐ No ☐ Not applicable
35. Has training been provided on infection prevention and PPE use for employees and/or volunteers?
☐ Yes, employees only ☐ Yes, employees and volunteers ☐ No
36. Is there an area for donning and doffing (putting on and taking off) PPE? ☐ Yes ☐ No ☐ Not applicable
37. Are there challenges in sourcing PPE? ☐ Yes ☐ No ☐ Don't know ☐ Not applicable

Instructions: This is the end of the facility questionnaire. Thank you for your time!

"Public reporting burden of this collection of information is estimated to average 10 minutes per response, including the time for reviewing instructions, searching existing data sources, gathering and maintaining the data needed, and completing and reviewing the collection of information. An agency may not conduct or sponsor, and a person is not required to respond to a collection of information unless it displays a currently valid OMB control number. Send comments regarding this burden estimate or any other aspect of this collection of information including suggestions for reducing this burden to CDC/ATSDR Reports Clearance Officer; 1600 Clifton Road NE, MS D-74 Atlanta, Georgia 30333; ATTN: PRA (0920-1011)."

Figure S2: Individual Animal Questionnaire for Shelter Animals Exposed to Persons with COVID-19

Instructions: A separate, individual animal questionnaire should be completed by an adult (≥ 18 years old) representative from each facility for each companion animal within the facility that may have had contact with a COVID-19 positive human case.

Facility ID: _________
CDC-assigned Animal ID: _________
State/Local Health Department:______________________________

Date interview completed: / / (MM/DD/YYYY)
Type of interview: ☐ On-site (in person) ☐ Off-site (by phone) ☐ Other (specify): __________________________________
Interviewer name and organization: __________________________________
Respondent's name: __________________________________
Respondent's position at the facility: __________________________________
Respondent's phone number: __________________________________

Prompt: All questions and answers in this survey will remain confidential. The first section asks questions about the individual animal. Please answer to the best of your ability.

38. Animal name: ____

39. Who owns the animal?: ☐ Private citizen (plus court holds) ☐ Facility/municipality ☐ Stray hold ☐ Don't know ☐ Other (specify): ____________________________
    a. If owned by a private citizen, has proper consent from the owner been obtained? ☐ Yes ☐ No

40. Shelter-assigned animal ID (e.g., intake ID): _________________

41. Animal species: ☐ Dog ☐ Cat ☐ Ferret ☐ Rabbit ☐ Other (please describe) ______________________________
    a. Breed/Description: ____

42. Age of the animal: ______________ years/months (circle one)

43. Sex of the animal: ☐ Male, intact ☐ Male, neutered ☐ Female, intact ☐ Female, spayed

44. Has the animal received any of the following vaccinations? (check all that apply):

Vaccinations	Last date administered (MM/DD/YYYY)
☐ Rabies	
☐ Feline Infectious Peritontis (FIP)/ Feline coronavirus (FCoV) coronavirus (FIP)	
☐ Canine coronavirus	
☐ Don't know	n/a
☐ None	n/a

45. Did the animal have any health conditions prior to coming into contact with the staff diagnosed with COVID-19? ☐ Yes, one condition ☐ Yes, two conditions ☐ Yes, 3 or more conditions ☐ No ☐ Don't know
    a. If yes, describe the conditions and their start date: ____
    ____
    b. If yes, is the animal on any medications or supplements for these conditions?: ____

46. Since coming into contact with the person(s) or animal(s) diagnosed with SARS-CoV-2 infection, has the animal developed any of the following symptoms?

Condition	Date of Onset (MM/DD/YYYY)	Date of Resolution (MM/DD/YYYY)
☐ Coughing	____/____/______ ☐ Date unknown	____/____/______ ☐ Not resolved
☐ Sneezing	____/____/______ ☐ Date unknown	____/____/______ ☐ Not resolved

"Public reporting burden of this collection of information is estimated to average 10 minutes per response, including the time for reviewing instructions, searching existing data sources, gathering and maintaining the data needed, and completing and reviewing the collection of information. An agency may not conduct or sponsor, and a person is not required to respond to a collection of information unless it displays a currently valid OMB control number. Send comments regarding this burden estimate or any other aspect of this collection of information including suggestions for reducing this burden to CDC/ATSDR Reports Clearance Officer; 1600 Clifton Road NE, MS D-74 Atlanta, Georgia 30333; ATTN: PRA (0920-1011)."

☐ Runny nose	____/____/______ ☐ Date unknown	____/____/______ ☐ Not resolved
☐ Eye discharge	____/____/______ ☐ Date unknown	____/____/______ ☐ Not resolved
☐ Difficulty breathing or shortness of breath	____/____/______ ☐ Date unknown	____/____/______ ☐ Not resolved
☐ Lethargy	____/____/______ ☐ Date unknown	____/____/______ ☐ Not resolved
☐ Vomiting	____/____/______ ☐ Date unknown	____/____/______ ☐ Not resolved
☐ Diarrhea	____/____/______ ☐ Date unknown	____/____/______ ☐ Not resolved
☐ Other (please describe): __________________________	____/____/______ ☐ Date unknown	____/____/______ ☐ Not resolved

47. Has any diagnostic testing been performed related to these new symptoms? ☐ Yes ☐ No ☐ Don't know
    a. If yes, what testing was performed? ____

48. Has the animal been treated for these new symptoms? ☐ Yes ☐ No ☐ Don't know ☐ Not applicable
    a. If yes, what treatments were given? ____
    b. If treatments given, what did they start and end? ______________________________________

49. What was the animal's shelter intake date (most recent): / / (MM/DD/YYYY)

50. What was the reason for the animal's intake?
    ☐ Surrender related to COVID-19 (e.g., illness or death of owner from COVID-19)
    ☐ Owner surrender unrelated to COVID-19
    ☐ Stray/loose animal (includes animals picked up by staff in the field and animals brought to the facility by the public)
    ☐ Confiscation/Cruelty
    ☐ Bite hold
    ☐ Returned by adopter or foster
    ☐ Transferred from another facility (e.g. animal shelter)
    ☐ Other, specify: ______________________
    ☐ Don't know

    a. If the animal was surrendered due to COVID-19, indicate why:
        ☐ Owner or member of family passed away from COVID-19
        ☐ Owner or member of family is ill with COVID-19
        ☐ Financial concerns related to COVID-19
        ☐ Concerns related to animals spreading the virus that causes COVID-19
        ☐ Don't know
        ☐ Other, specify: ______________________

The next section asks some additional questions related to how the animal may have been exposed to SARS-CoV-2 within the facility.

51. How was the animal exposed to SARS-CoV-2? (mark all that apply)
    ☐ Shelter employee (paid or unpaid)
    ☐ Shelter volunteer
    ☐ Shelter visitor (e.g., rescue group)
    ☐ Previous or current owner
    c Another pet in the previous home (specify): ______________________
    ☐ Shelter cat

"Public reporting burden of this collection of information is estimated to average 10 minutes per response, including the time for reviewing instructions, searching existing data sources, gathering and maintaining the data needed, and completing and reviewing the collection of information. An agency may not conduct or sponsor, and a person is not required to respond to a collection of information unless it displays a currently valid OMB control number. Send comments regarding this burden estimate or any other aspect of this collection of information including suggestions for reducing this burden to CDC/ATSDR Reports Clearance Officer; 1600 Clifton Road NE, MS D-74 Atlanta, Georgia 30333; ATTN: PRA (0920-1011)."

☐ Shelter dog
☐ Other, specify: ____
☐ Don't know

52. What was the duration of interaction between the animal and person(s)/animal(s) confirmed to have COVID-19?
☐ < 1 hr ☐ 1-3 hours ☐ 4-6 hours ☐ 7-9 hours ☐ 10-12 hours ☐ 12+ hours ☐ Don't know

53. When was last time the animal was exposed to a <u>person</u> with SARS-CoV-2? Please give your best estimate: / / (MM/DD/YYYY) ☐ Don't know

54. Types of interaction/contact with the human cases (mark all that apply):

☐ Going for walks or play yards	☐ Petting/cuddling	☐ Behavioral assessments or training
☐ Grooming	☐ Playgroups with other animals	☐ In staff member's office full time
☐ Feeding	☐ Veterinary care	☐ Cleaning the animal's housing
☐ Other (please describe):	☐ Don't know	

55. Did you observe any of the persons confirmed with COVID-19 doing any of the following when they were within six feet of the animal?
☐ Coughing
☐ Sneezing
☐ Exhibiting shortness of breath
☐ Other, specify: _____________
☐ Did not observe

56. When was last time the animal was exposed to an <u>animal</u> confirmed with SARS-CoV-2? Please give your best estimate: / / (MM/DD/YYYY) ☐ Don't know

57. Types of interaction/contact with the animal cases (mark all that apply):

☐ Going for walks within 6 ft of each other	☐ Housed in the same room, but separate kennels	☐ Sniffing each other in passing (e.g. between kennel door)
☐ Share communal housing	☐ Playing together in the same enclosure	☐ Visiting the same locations
☐ Sharing food/water bowls	☐ Sharing toys	☐ Don't know
☐ Other (please describe):		

58.

Prompt: This is the last section. It asks questions about the animal's housing and routines.

59. What is the room ID for the room/area where the animal is currently housed? _____________________________

60. What is the room ID for the room/area where the animal was potentially exposed? _________________________

61. Is the animal currently housed in an area designated for animals exposed to SARS-CoV-2 (e.g., isolation or quarantine room)?
☐ Yes, this area <u>only</u> houses animals exposed to SARS-CoV-2
☐ Yes, this is a general quarantine area that houses animals exposed to SARS-CoV-2 as well as animals with other medical conditions or exposures
☐ No ☐ Don't know

"Public reporting burden of this collection of information is estimated to average 10 minutes per response, including the time for reviewing instructions, searching existing data sources, gathering and maintaining the data needed, and completing and reviewing the collection of information. An agency may not conduct or sponsor, and a person is not required to respond to a collection of information unless it displays a currently valid OMB control number. Send comments regarding this burden estimate or any other aspect of this collection of information including suggestions for reducing this burden to CDC/ATSDR Reports Clearance Officer; 1600 Clifton Road NE, MS D-74 Atlanta, Georgia 30333; ATTN: PRA (0920-1011)."

62. Describe the animal's current housing:
   - ☐ Cohoused with other animals (e.g., communal cat room, multiple dogs in one run)
   - ☐ Housed individually, with other animals in the same room (e.g., a kennel with multiple, single animal enclosures in one room)
   - ☐ Housed individually, no other animals in the room (e.g., in a staff member's office)
   - ☐ Free-roaming outdoors (community cats)
   - ☐ Don't know ☐ Other, specify: ________________

63. Currently, does the animal leave their housing to interact or play with other animals (e.g., dogs participating in play group)? ☐ Yes, specify: __________________ ☐ No ☐ Don't know

64. Has the animal left the shelter since being exposed to SARS-CoV-2? ☐ Yes ☐ No ☐ Don't know
   a. If yes, why?
   - ☐ Adopted/fostered and returned
   - ☐ Community or volunteer event (e.g., an adoption event, taken out by a volunteer ("dog for the day"))
   - ☐ Walk outside of the shelter grounds
   - ☐ External veterinary services
   - ☐ Other, specify: _______________

65. On a regular day before the exposure to SARS-CoV-2 was recognized, how many shelter staff or volunteers interacted with the animal per day?
   ☐ 1 ☐ 2 ☐ 3 ☐ 4 ☐ 5 or more ☐ Don't know

66. On a regular day before the exposure to SARS-CoV-2 was recognized, how long per day and what types of interaction (e.g., walking, grooming, petting, cuddling) did the animal usually have with shelter staff?
   b. Duration of interaction with pet per day:
      ☐ <1 hour ☐ 1-3 hours ☐ 4-6 hours ☐ 7-9 hours ☐ 10-12 hours ☐ 12+ hours
   c. Types of interaction/contact with shelter staff (mark all that apply):

- ☐ Going for walks or play yards
- ☐ Grooming
- ☐ Feeding
- ☐ Other (please describe):
- ☐ Petting/cuddling
- ☐ Playgroups with other animals
- ☐ Veterinary care
- ☐ Behavioral assessments or training
- ☐ In staff member's office full time
- ☐ Cleaning the animal's housing

67. On a regular day after the exposure to SARS-CoV-2, how many shelter staff or volunteers interact with the animal per day? ☐ 1 ☐ 2 ☐ 3 ☐ 4 ☐ 5 or more

68. On a regular day since the animal was exposed to SARS-CoV-2, how long per day and what types of interaction has the animal had with shelter staff?
   d. Duration of interaction with pet per day:
      ☐ <1 hour ☐ 1-3 hours ☐ 4-6 hours ☐ 7-9 hours ☐ 10-12 hours ☐ 12+ hours
   e. Types of interaction/contact with pet (mark all that apply):

- ☐ Going for walks or play yards
- ☐ Grooming
- ☐ Feeding
- ☐ Other (please describe):
- ☐ Petting/cuddling
- ☐ Cleaning/disinfecting housing
- ☐ Veterinary care
- ☐ Behavioral assessments or training
- ☐ In staff member's office full time
- ☐ Cleaning the animal's housing

69. On a regular day after the animal was exposed to SARS-CoV-2, what personal protective equipment (PPE) do staff wear when providing basic care such as feeding and cleaning the animal's housing? (mark all that apply)
   - ☐ None
   - ☐ Protective outerwear (gown, coveralls)
   - ☐ Gloves
   - ☐ Eye protection (face shield, goggles)
   - ☐ Face mask
   - ☐ Respirator (N95 or alternative)

70. Is there any additional information you think we should know about the animal?
   a. If yes: ____

Instructions: You have completed the questionnaire for this animal. Thank you for your time and participation!

"Public reporting burden of this collection of information is estimated to average 10 minutes per response, including the time for reviewing instructions, searching existing data sources, gathering and maintaining the data needed, and completing and reviewing the collection of information. An agency may not conduct or sponsor, and a person is not required to respond to a collection of information unless it displays a currently valid OMB control number. Send comments regarding this burden estimate or any other aspect of this collection of information including suggestions for reducing this burden to CDC/ATSDR Reports Clearance Officer; 1600 Clifton Road NE, MS D-74 Atlanta, Georgia 30333; ATTN: PRA (0920-1011)."
